# Supplementary material for: Sophorolipid Suppresses LPS-Induced Inflammation in RAW264.7 Cells through the NF-κB Signaling Pathway
Source: Molecules. 2022 Aug 8;27(15):5037. doi: 10.3390/molecules27155037 (PMC9370320; doi:10.3390/molecules27155037)
Supplement: Supplementary file 1 [file molecules-27-05037-s001.zip › molecules-1834928-supplementary.pdf]

Sophorolipid suppresses LPS-induced inflammation in RAW264.7 cells through NF-κB signaling pathway

Rui-Qi Xu[1,†], Ling Ma[2,†], Timson Chen[2], Jing Wang[1,*]

[1] Key Laboratory of Synthetic and Biological Colloids, Ministry of Education, School of Chemical and Material Engineering, Jiangnan University, Wuxi 214122, China

[2] Adolph Innovation Laboratory, Guangzhou Degu Personal Care Products Co., Ltd., Guangzhou 510000, China

[*] Correspondence: jingwang@jiangnan.edu.cn

[†] These authors contributed equally to this work.

Liquid chromatography - mass spectrometry:

Acidic components (R.T- 0.5-4.0)are eluted first while the lactonic (R.T- 3.0-10.0) are eluted later due to their higher hydrophobicity.

The following figures depict the liquid chromatography and mass spectra of SL we got: to identify the presence of different modifications. Figure S1(a) reveals the mass spectrum of SL with molecular formula $C_{34}H_{56}O_{14}$. The spectrum shows presences of amine adduct at m/z 706 and sodium adduct at m/z 711 respectively.

(a)

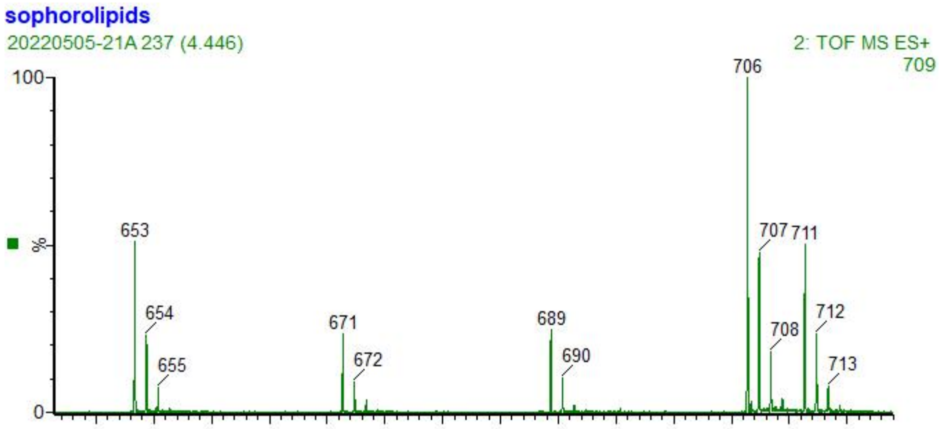

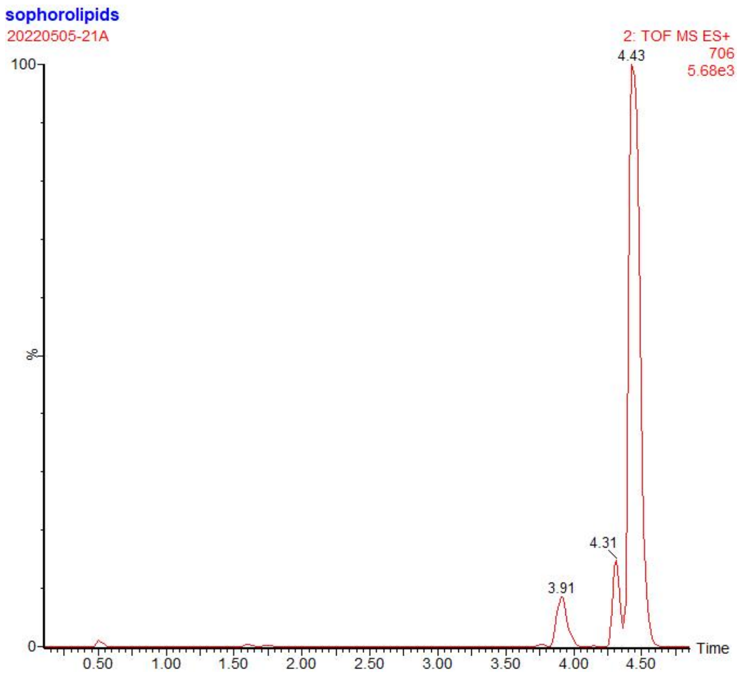

(b)

Figure S1 (a): Mass spectrum of di-acetylated lactonic form S1 (b): Liquid chromatography of di-acetylated lactonic form

Figure S2(a) reveals the mass spectrum of SL with molecular formula $C_{34}H_{58}O_{15}$ and $C_{34}H_{60}O_{15}$. The spectrum shows presences of sodium adduct at m/z 729、731 and amine adduct at m/z 724 respectively. The mass spectrum also shows presence of protonated molecular ion peak at m/z 707.

(a)

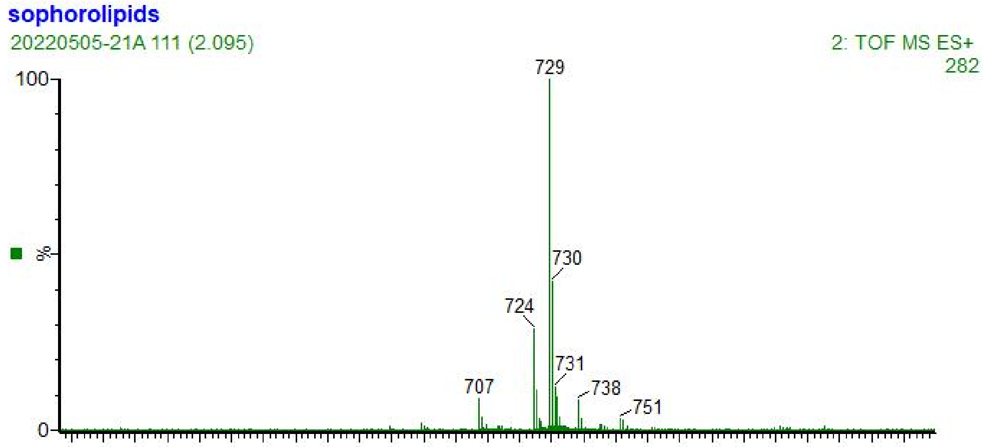

(b)

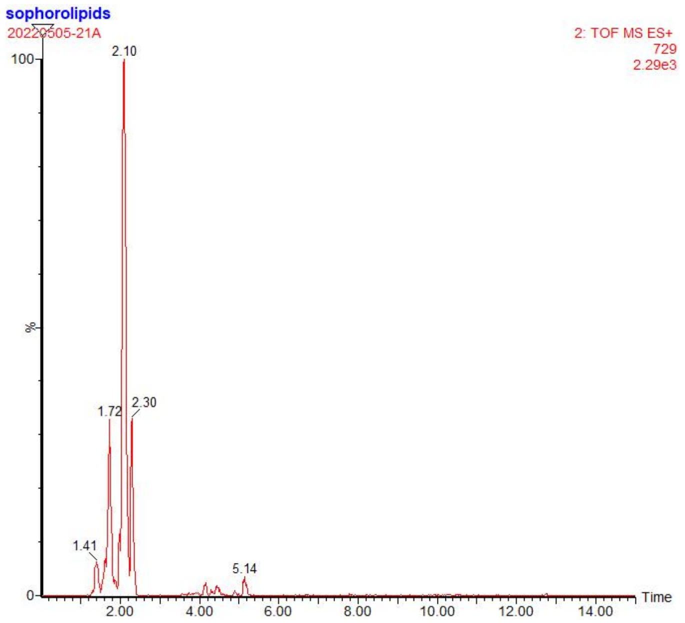

Figure S2 (a): Mass spectrum of di-acetylated acidic form S2 (b): Liquid chromatography of di-acetylated acidic form

Figure S3(a) reveals the mass spectrum of SL with molecular formula $C_{34}H_{58}O_{14}$. The spectrum shows presences of amine adduct at m/z 708 and sodium adduct at m/z 713 respectively.

(a)

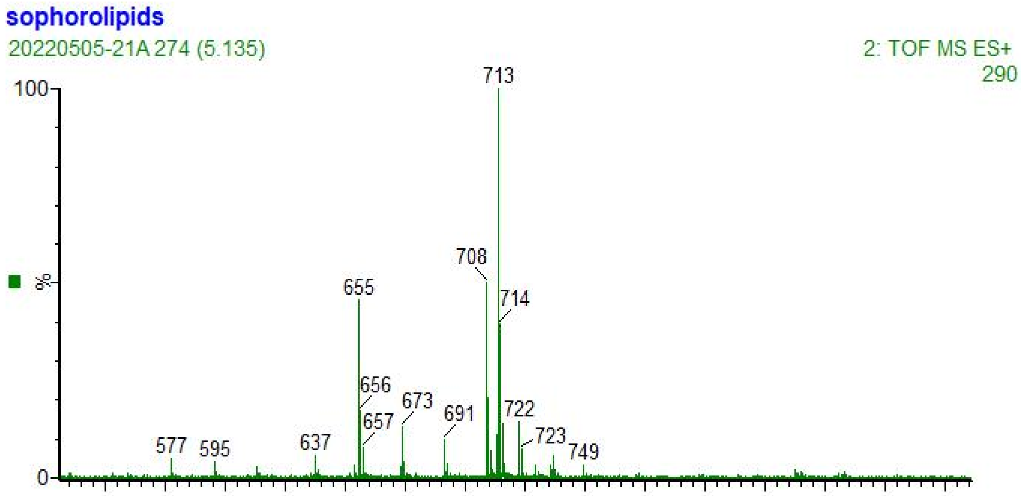

(b)

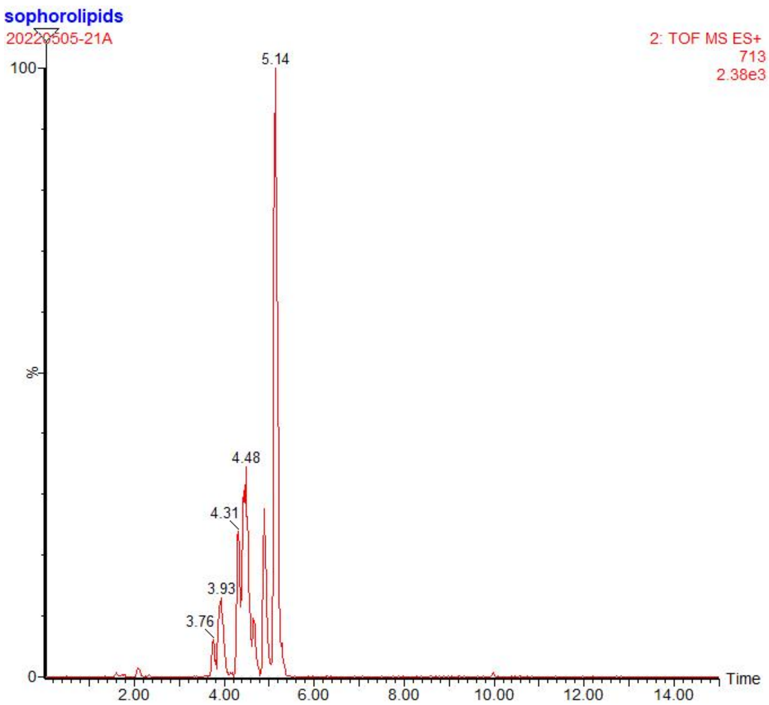

Figure S3 (a): Mass spectrum of di-acetylated lactonic form S3 (b): Liquid chromatography of di-acetylated lactonic form

Figure S4(a) reveals the mass spectrum of SL with molecular formula $C_{34}H_{56}O_{15}$.The spectrum shows presences of sodium adduct at m/z 727 and amine adduct at m/z 722 respectively. The mass spectrum also shows presence of deprotonated molecular ion peak at m/z 703.

(a)

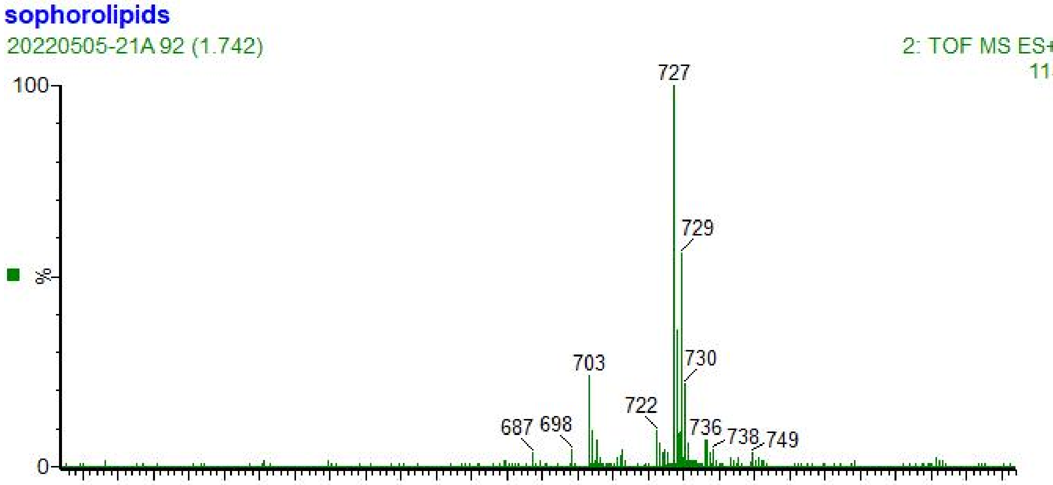

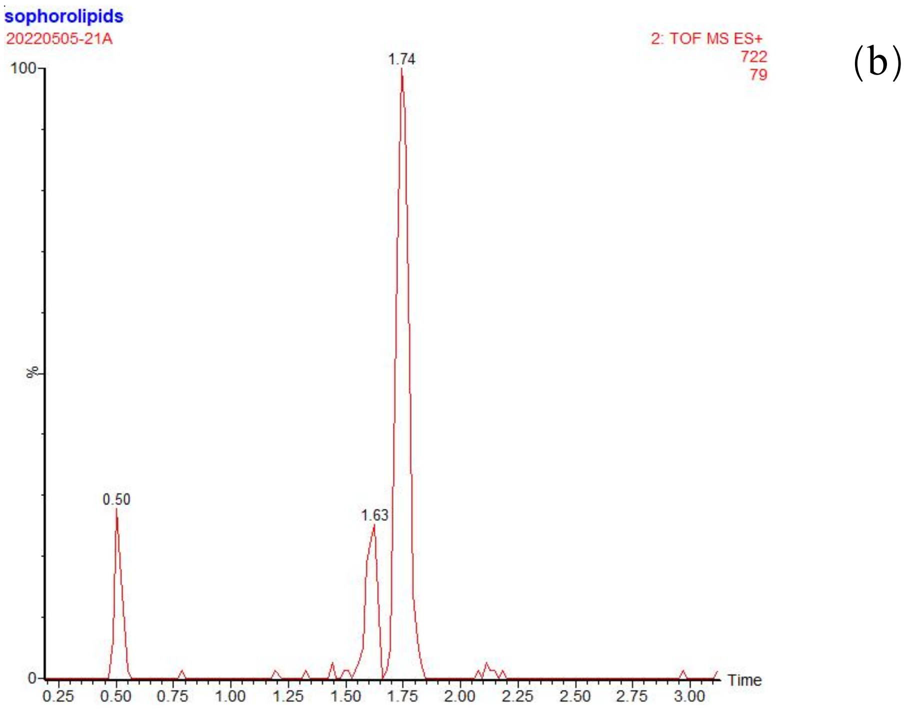

Figure S4 (a): Mass spectrum of di-acetylated acidic form S4 (b): Liquid chromatography of di-acetylated acidic form

Figure S5(a) reveals the mass spectrum of SL with molecular formula $C_{34}H_{54}O_{14}$. The spectrum shows presences of amine adduct at m/z 708 and sodium adduct at m/z 713 respectively.

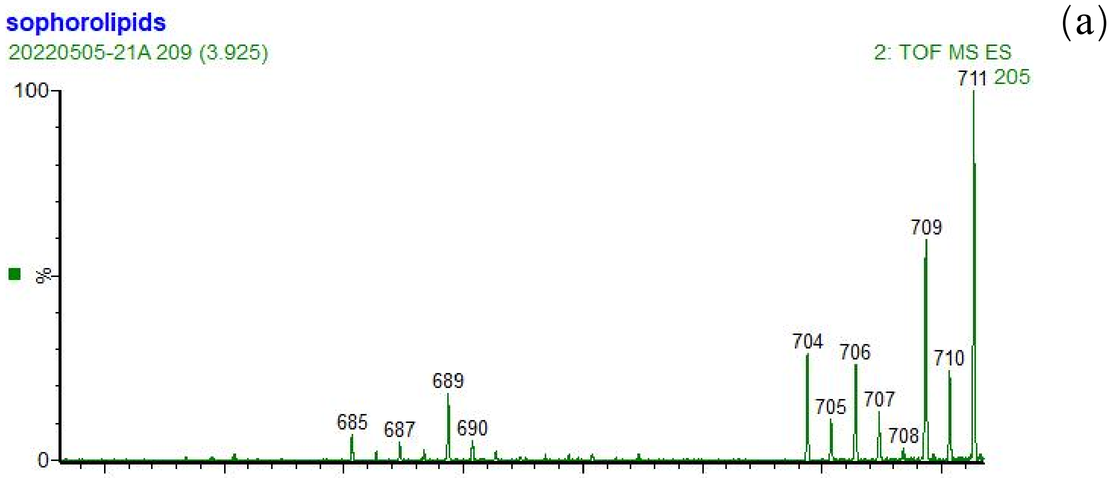

(b)

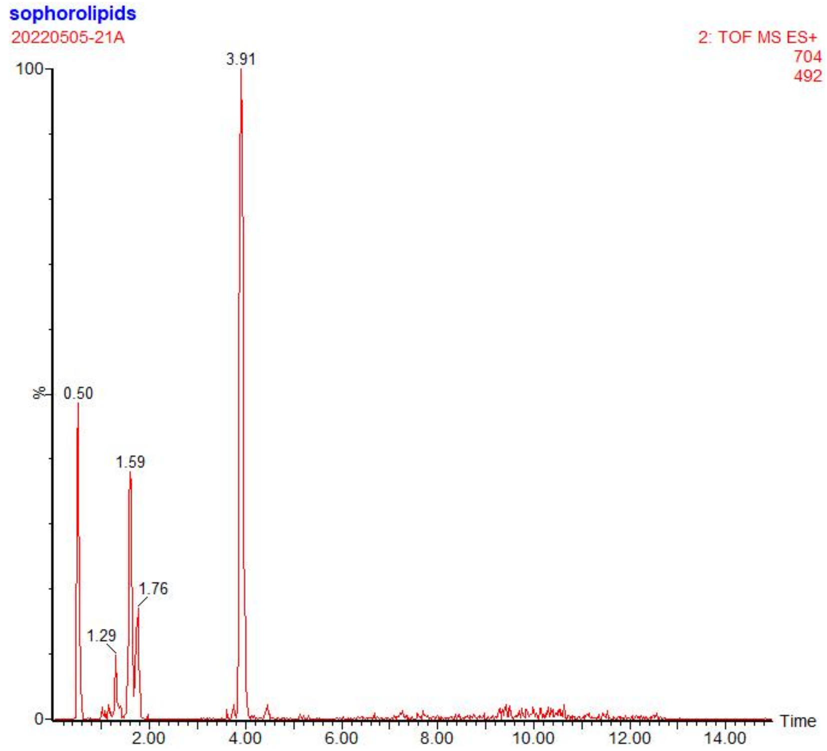

Figure S5 (a): Mass spectrum of di-acetylated lactonic form S5 (b): Liquid chromatography of di-acetylated lactonic form

Figure S6(a) reveals the mass spectrum of SL with molecular formula $C_{34}H_{54}O_{13}$. The spectrum shows presences of amine adduct at m/z 664 and sodium adduct at m/z 669 respectively.

(a)

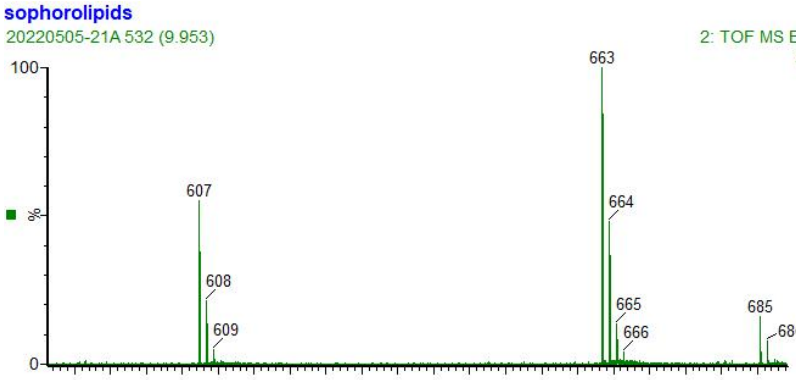

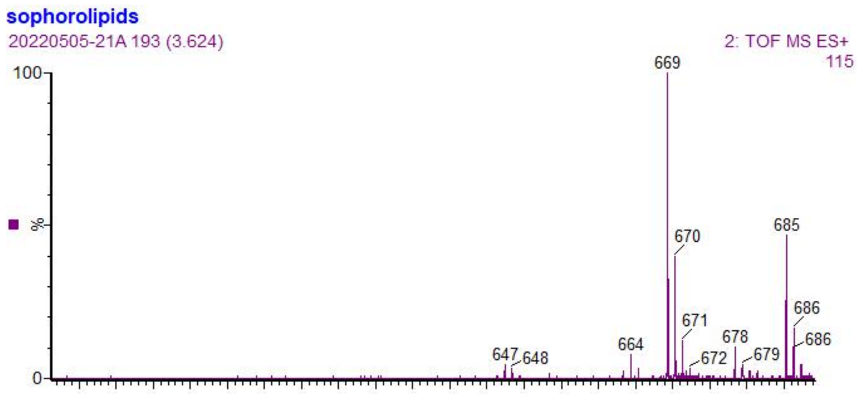

(b)

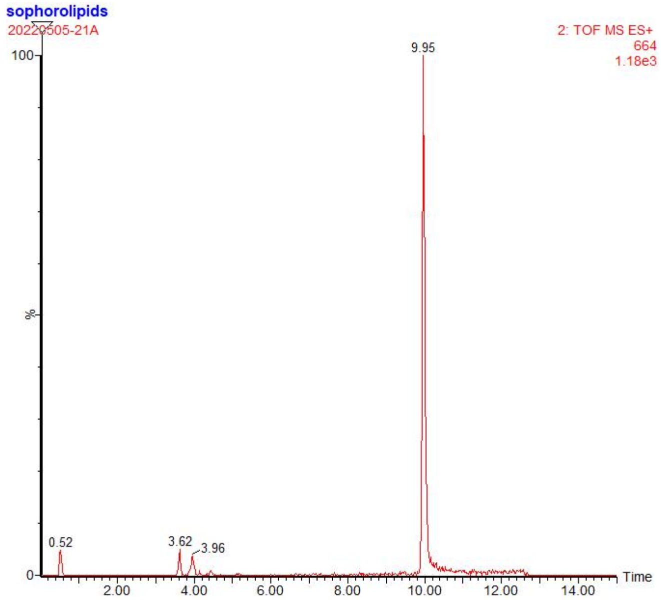

Figure S6 (a): Mass spectrum of mona-acetylated lactonic form S6 (b): Liquid chromatography of mona-acetylated lactonic form

Figure S7(a) reveals the mass spectrum of SL with molecular formula $C_{34}H_{56}O_{13}$. The spectrum shows presences of sodium adduct at m/z 671.

(a)

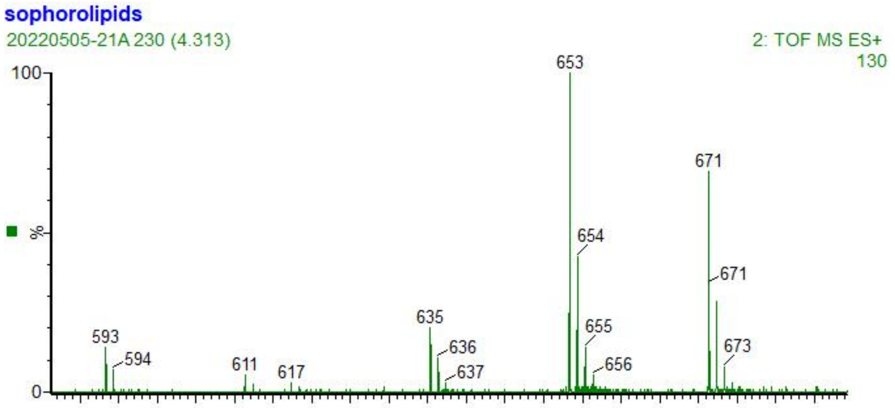

(b)

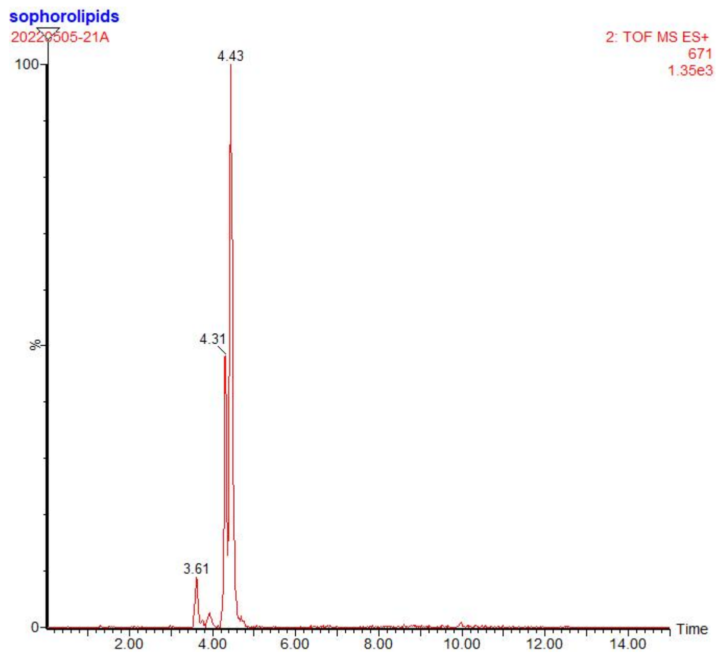

Figure S7 (a): Mass spectrum of mona-acetylated lactonic form S7 (b): Liquid chromatography of mona-acetylated lactonic form

Figure S8(a) reveals the mass spectrum of SL with molecular formula $C_{32}H_{54}O_{14}$.The spectrum shows presences of amine adduct at m/z 680.

(a)

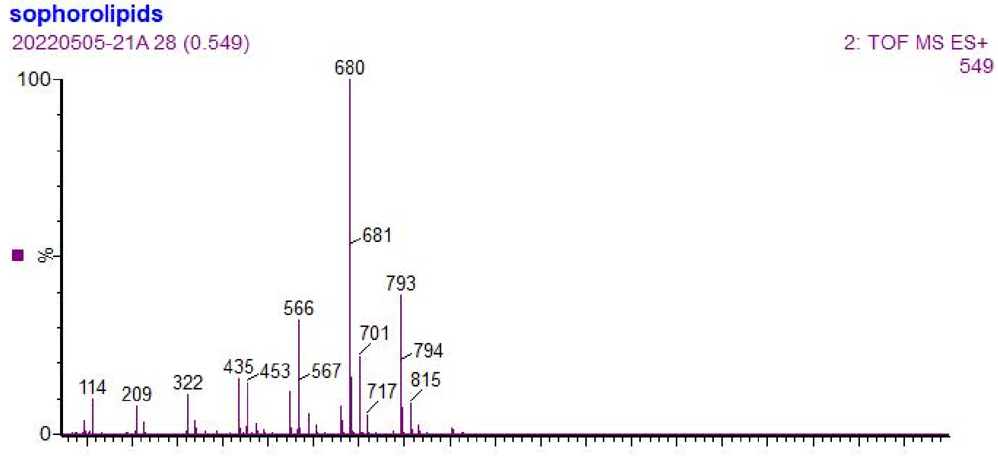

(b)

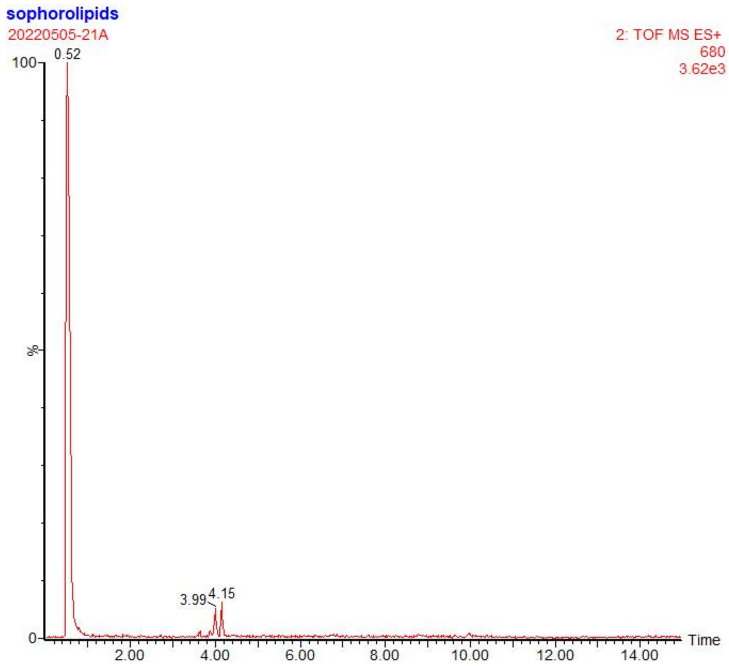

Figure S8 (a): Mass spectrum of mona-acetylated acidic form S8 (b): Liquid chromatography of mona-acetylated acidic form
